# Colloidal Nanomolybdenum Influence upon the Antioxidative Reaction of Chickpea Plants (*Cicer arietinum* L.)

**DOI:** 10.1186/s11671-016-1690-4

**Published:** 2016-10-26

**Authors:** Nataliya Taran, Ludmila Batsmanova, Oksana Kosyk, Oleksandr Smirnov, Mariia Kovalenko, Liubov Honchar, Alexander Okanenko

**Affiliations:** 1 Institute of Biology, Taras Shevchenko National University of Kyiv, 64/13, Volodymyrska Street, Kyiv, 01601 Ukraine; 2 National University of Life and Environmental Sciences of Ukraine, 12 Heroiv Oborony, Kyiv, 03041 Ukraine

**Keywords:** Nanoparticles, Molybdenum, Chickpea, Microorganisms, Catalase, Superoxide dismutase

## Abstract

The use of colloidal solutions of metals as micronutrients enhances plant resistance to unfavorable environmental conditions and ensures high yields of food crops. The purpose of the study was a comparative evaluation of presowing treatment with nanomolybdenum and microbiological preparation impact upon the development of adaptive responses in chickpea plants. Oxidative processes did not develop in all variants of the experiment but in variants treated with microbial preparation, and joint action of microbial and nanopreparations even declined, as evidenced by the reduction of thiobarbituric acid reactive substances in photosynthetic tissues by 15 %. The activity of superoxide dismutase increased (by 15 %) in variant “nanomolybdenum” and joint action “microbial + nanomolybdenum,” but it decreased by 20 % in variants with microbial preparation treatment. The same dependence was observed in changes of catalase activity. Antioxidant status factor, which takes into account the ratio of antioxidant to pro-oxidant, was the highest in variants with joint action of microbial preparation and nanomolybdenum (0.7), the lowest in variants with microbial treatment only (0.1). Thus, the results show that the action of nanoparticles of molybdenum activated antioxidant enzymes and decreased oxidative processes, thus promoting adaptation of plants.

## Background

Plants contain molybdenum in small quantities (0.001–0.1 mg% in terms of dry matter), but it has an important role in phosphorus and protein metabolism. Molybdenum is present in all organs, part of the 20 enzymes (aldehyde oxidases, hydrogenases, nitrate reductase) that catalyze the transition of nitrates into nitrites. It should be noted particularly for its role in the metabolism of legumes because molybdenum is involved in fixing of molecular nitrogen by nodule bacteria of the genus *Rhizobium* [1]. Formation of legume-rhizobial symbiosis includes a number of stages, where the enzyme complex, nitrogenase, is synthesized. It catalyzes the reduction of molecular nitrogen from the atmosphere [2]. This complex consists of two enzymes: the actual nitrogenase (so-called MoFe protein or dinitrogenase) and dehydrogenase (Fe protein) [3]. The MoFe protein cofactor consists two atoms of molybdenum, which determine the influence of nanomolybdenum colloidal solution on nodulation—central link of legume—and rhizobial symbiosis, providing the necessary conditions for the formation and functioning of the enzyme complex and nitrogen-fixing system [2, 4]. Now, the great interest to the group of biologically active substances—nanosolution of metals (colloidal solutions of metals), owing to the structurally phased condition, gets a set of properties useful to biological objects. The nanoparticles of metals can be the activators of antioxidant protective mechanisms of plants while cultivating in various stressful conditions. There is no single opinion on the impact of metal nanoparticles upon physiological and biochemical processes in plants in available literature—as positive and negative effects are noted. Our previous studies have shown change in element metal content in roots and shoots of winter wheat plants at colloidal solution nanosized particles of metal (Cu, Zn, Fe, Mn) action [5, 6]. Also, we got a positive nanomolybdenum impact upon the root microflora *Cicer arietinum* L. [7]. Nowadays, electrospark technologies for obtaining nanostructured metals and alloys are the most effective and can meet the requirements of scientific and applied problems [8].

A colloidal form of metal nanosized particles that have been derived from underwater electric discharge between the conductive granules was used in our studies. Dispersed phase is formed depending upon the discharge circuit in two size ranges—micro- and nanometer ranges. Micro fraction is the result of melting the surface of metal pellets, followed by crystallization; due to its large size, it cannot be used as an effective form of trace elements for use in biological objects. With nanofraction arising from melting and evaporation followed by vapor condensation of average size in the range of 10–150 nm, corresponding structural and phase composition of the solid phase has signs of biological function and can be used in biotechnology, particularly in the technologies of growing vegetable products. With the increase of nanoparticle size or aggregate formation, as well as with increasing degree of oxidation of the metal phase (formation of CuO, Fe_3_O_4_, and MnO_2_ oxides) and amount of oxide phase on particle surface, their biological activity slowed down [9]. A metastable non-equilibrated nanoparticle state cannot allow to predict the physiological and biochemical processes in plants; therefore, these studies remain actual today.

Taking into account the widespread use of microbiological origin preparation to improve the nitrogen fixation of plants, the purpose of our study was a comparative assessment of nanomolybdenum and microbiological preparations of *Ryzobofitom* impact upon the development of adaptive responses in chickpea plants. Chickpea plants are drought-tolerant and are able to fix atmospheric nitrogen by forming the symbiotic relationships with nitrogen fixation microorganisms that not only meet the requirements of plants in nitrogen but also bring it into the ground [10].

## Methods

The object of the study was the chickpea plant (*Cicer arietinum* L.) of the Rosanna middle ripening variety. The investigation was performed in field conditions. The soil of the experimental field was typical black soil with 4.38–4.53 % humus content in the arable soil layer, pH of salt extract—6.9–7.3, nitrogen—0.27–0.31 %, phosphorus—0.15–0.25 %, and potassium—2.3–2.5 %. Farming equipment is common to the northern forest-steppe of Ukraine. Presowing treatment of plants with colloidal solution of molybdenum nanoparticles (1 l—per ton of seeds, working solution measured up metal concentration of 0.8 mg/g) and microbiological preparations of *Ryzobofitom* (1 ml contained 6.7 billion nodule bacteria of the genus *Rhizobium*) was performed. Nanomolybdenum was received by electric-sparkle dispersing of the electric-conductive layer in the water. Submicron metal particles in water suspensions were obtained by the method of volumetric electric-spark destruction of metal granules. The pulse power source (thyristor pulse generators with the storage capacitor) was used to initiate the discharge and simultaneous formation of spark channels in contacts between the metal granules dipped into the deionized water. The melting of some surface areas take place during the electrical erosion of metal granules with the formation of the metal fraction with a diameter of 10 to 500 μm. The transformation of liquid to vapor and its condensation with following crystallization results in the creation of fraction with size from 10 to 100 nm. Based on the fact that the fundamental properties of nanomaterials are determined at the stage of their formation, their efficiency is connected with kinetics, the processes occurring in the discharge chamber reaction zone. Joint action of intense heat and force on the metal taking place during the ultrashort time intervals results in obtaining nanomaterials with a non-equilibrium structure and increased level of free energy [11]. This method allowed achieving a dispersed-isolated state of the solid phase and the formation of colloid systems with satisfactory aggregative stability associated with biological functionality of metal colloidal solutions. Colloidal solution of molybdenum nanoparticles was obtained by means of metal dispersion by electric current pulses with an amplitude of 100 to 2000 A in water [12]. With this, the master colloidal solution contains an aqueous solution of molybdenum nanoparticles with a concentration of not less than 8 mg/l. The size of nanoparticles of metals is from 100 to 250 nm, and their concentration in bidistilled water is not more than the value calculated by Eq. 1.

1 $$ m\le 1278\times V-0.8, $$

where *m* is the concentration of nanoparticles of metal (mg/l) and *V* is the volume of one mole of metal atoms (cm^3^/mol). The colloidal solution of nanoparticles of molybdenum was used in the dose of 1 μl per gram (μl/g).

Treatment was performed according to following scheme: (1) control and treatment of water, (2) presowing treatment with molybdenum nanoparticles, (3) presowing treatment with microbiological preparation of *Ryzobofitom*, and (4) presowing treatment with molybdenum nanoparticles and microbiological preparation of *Ryzobofitom*.

### Enzymatic Activity

Extraction of antioxidant enzymes was performed according to Rios-Gonzalez et al. [13]. The activity of superoxide dismutase (SOD; EC 1.15.1.1) was determined by the ability to inhibit recovery of nitroblue tetrazolium (NBT) at *λ* = 560 nm. The reaction mixture containing 1 ml of riboflavin, 1 ml of methionine, 1 ml NBT, and 50 ml of extract. The unit of enzyme activity was 50 % inhibition of formazan formation. SOD activity is expressed in arbitrary units per mg protein extract [14].

The activity of catalase (CAT; EC 1.11.1.6) was determined according to Aeby [15]. The reaction mixture contained 2.9 ml of phosphate buffer (pH 7.0), 90 ml of extract, and 10 ml of 33 % H_2_O_2_. Measurements were carried out at *λ* = 240 nm for 60 s. Activity was expressed as arbitrary (arb.) units per mg of protein.

### Determining the Level of Lipid Peroxidation

The level of lipid peroxidation was determined by the accumulation of thiobarbituric acid reactive substances (TBARS) according to the method of Kumar and Knowles [16] with modifications. Two hundred milligrams of photosynthetic tissues was homogenized with a small amount (1.5 ml) of Tris-NaCl buffer (pH 7.6), and the volume of the extract was adjusted to 3 ml. To the resulting homogenate was added 1 ml of 0.5 % solution thiobarbituric acid (TBA) and 2 ml of 20 % trichloroacetic acid (TCA). Test tubes with homogenates withstand 30 min in a boiling water bath, followed by centrifugation at 3000 rev/min for 10 min. Determination was carried out at *λ* = 533 nm. Quantity of TBARS is expressed in micromoles of malonic dialdehyde using molar extinction coefficient 1.55 × 10^5^ cm^−1^ M^−1^. Integral indicator of overall antioxidant activity was calculated according to Eq. 2.

2 $$ F=\left(\mathrm{C}\mathrm{A}{\mathrm{T}}_{\mathrm{a}}\times \mathrm{SO}{\mathrm{D}}_{\mathrm{a}}\right)/\mathrm{TBAR}{\mathrm{S}}_{\mathrm{con}}, $$

where *F* is the factor antioxidant status, CAT_a_ is the CAT activity, SOD_a_ is the SOD activity, and TBARS_con_ is the TBARS content [17] characterizing antioxidant potential.

Chlorophylls *a*, *b*, and carotenoid content were determined by measuring alcohol extract optical density [18]. Determination of protein was performed according to Bradford [19]. The reaction mixture contained 2 ml of Bradford reagent, 16 ml of 0.15 M NaCl and 40 ml of extract. The tubes were left for 2 min; optical density was measured on a spectrophotometer UV-1800 (Shimadzu) at *λ* = 595 nm. Protein concentration of the extract was calculated according to the calibration curve for bovine albumin.

Statistical analysis of the data was made using program Statistica 6.0. Probability of the difference between the arithmetic mean of indicators was established using Student’s test. The differences are considered to be significant at a value *P* ≤ 0.05 [20].

## Results and Discussion

**Fig. 1:**
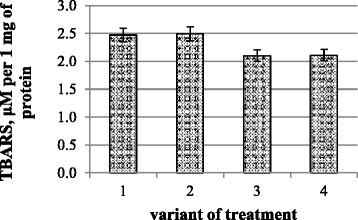
The content of TBARS in chickpea plant photosynthetic tissues: (*1*) control and water treatment, (*2*) presowing treatment with nanoparticles of molybdenum, (*3*) presowing treatment with microbiological preparation of *Ryzobofitom*, and (*4*) presowing treatment with nanoparticles of molybdenum and microbiological preparation of *Ryzobofitom*

Analysis of the results showed that oxidative processes are not developed in all variants of the experiment, and in variants with action of microbial preparation (variant 3), but joint action of nanomolybdenum and microbial preparation (variant 4) caused decrease in their activity, as evidenced by the reduction of TBARS in photosynthetic tissues by 15 % (Fig. 1).

**Fig. 2:**
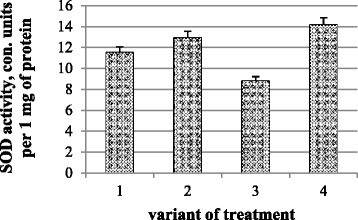
The activity of SOD in chickpea plant photosynthetic tissues: (*1*) control and water treatment, (*2*) presowing treatment with nanoparticles of molybdenum, (*3*) presowing treatment with microbiological preparation of *Ryzobofitom*, and (*4*) presowing treatment with nanoparticles of molybdenum and microbiological preparation of *Ryzobofitom*

SOD is an important component of the antioxidant defense system in plants and catalyzes dismutation of superoxide into oxygen and hydrogen peroxide. SOD activity is related to the intensity of lipid peroxidation and depends upon the number of accumulated intermediates. Analysis of the results showed that SOD activity increased (by 15 %) in variants with nanomolybdenum action and joint action of nanomolybdenum and microbial preparation, but in variants with only microbial preparation, it decreased by 20 % (Fig. 2).

**Fig. 3:**
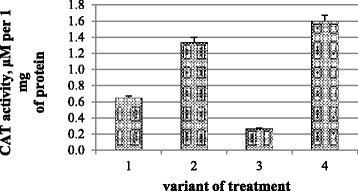
The activity of catalase in chickpea plant photosynthetic tissues: (*1*) control and water treatment, (*2*) presowing treatment with nanoparticles of molybdenum, (*3*) presowing treatment with microbiological preparation of *Ryzobofitom*, and (*4*) presowing treatment with nanoparticles of molybdenum and microbiological preparation of *Ryzobofitom*

A similar dependence was observed in changes of CAT activity: it grew in variants with nanomolybdenum and nanomolybdenum and microbial preparation joint treatments twice and 2.5 times, respectively, but it decreased by 2.5 times in variants with microbial preparation treatment only (Fig. 3).

Some investigators found a stimulating effect of metal nanoparticles upon chlorophyll content [21]. Others pay attention to chromosomal aberrations of young plants treated with magnetic nanoparticles, and the level of chlorophyll was increased at their low concentrations and decreased at higher concentrations [22].

**Fig. 4:**
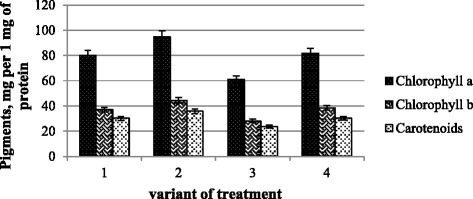
Pigment content: *Chl a* chlorophyll a, *Chl b* chlorophyll b, *Car* carotenoids (*1*) control and water treatment, (*2*) presowing treatment with nanoparticles of molybdenum, (*3*) presowing treatment with microbiological preparation of *Ryzobofitom*, and (*4*) presowing treatment with nanoparticles of molybdenum and microbiological preparation of *Ryzobofitom*

Our study showed a significant increase of photosynthetic pigment content at nanomolybdenum action (Fig. 4). The stimulating effect of nanoparticles may be due to their high reactivity and ability to form agglomerates and prolonged action at the tissue level [23].

In general, there is an increase in dynamics of chlorophyll *a* and chlorophyll *b* while maintaining stable levels of carotenoids or some reduction of their contents. We can assume that treatment with molybdenum nanoparticles contributed to the preservation of pool of chlorophylls by maintaining a certain level of carotenoids, which are able to quench an excited state of chlorophyll and prevent the formation of singlet oxygen and other reactive oxygen species (ROS) [24]. Joint action of nanomolybdenum and microbiological preparation keep stable these indexes appropriate to control variants, but the microbiological preparation treatment only (variant 3) declined them significantly.

## Conclusions

**Fig. 5:**
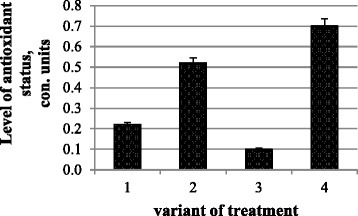
Factor of the antioxidant activity: (*1*) control and water treatment, (*2*) presowing treatment with nanoparticles of molybdenum, (*3*) presowing treatment with microbiological preparation of *Ryzobofitom*, and (*4*) presowing treatment with nanoparticles of molybdenum and microbiological preparation of *Ryzobofitom*

To characterize the adaptive responses of plants, the most prominent is factor of antioxidant activity (FAA), which takes into account the ratio of antioxidant to pro-oxidant (Fig. 5). It was highest in variants with joint action of nanomolybdenum and microbial preparation (0.7); the smallest was at microbial preparation action (0.1). Nanomolybdenum keeps FAA equal to 0.52—more than two times higher than in the control variants.

Thus, modification of microbial preparation with nanomolybdenum raised its bioactivity, probably due to the fact that metal nanoparticles have excess energy; they are highly reactive and can engage in the process of aggregation. In addition, they can bind to different organelles in cytoplasm thus changing intracellular and physiological features.

Thus, the results point to the indirect effect of molybdenum nanoparticles upon the metabolic processes that lead to activation of antioxidant enzymes and reduction of oxidative processes, thus promoting adaptation of plants.
